# Size matters in atrial fibrillation: the underestimated importance of reduction of contiguous electrical mass underlying the effectiveness of catheter ablation

**DOI:** 10.1093/europace/euab078

**Published:** 2021-05-05

**Authors:** Adam Hartley, Joseph Shalhoub, Fu Siong Ng, Andrew D Krahn, Zachary Laksman, Jason G Andrade, Marc W Deyell, Prapa Kanagaratnam, Markus B Sikkel

**Affiliations:** 1 National Heart and Lung Institute, Imperial College London, London, UK; 2 Division of Cardiology, University of British Columbia, 740 Hillside Ave, Vancouver, BC V8T 1Z4, Canada; 3 Division of Medical Sciences, University of Victoria, Victoria, Canada

**Keywords:** Atrial fibrillation, Catheter ablation, Critical mass, Atrial size

## Abstract

Evidence has accumulated over the last century of the importance of a critical electrical mass in sustaining atrial fibrillation (AF). AF ablation certainly reduces electrically contiguous atrial mass, but this is not widely accepted to be an important part of its mechanism of action. In this article, we review data showing that atrial size is correlated in many settings with AF propensity. Larger mammals are more likely to exhibit AF. This is seen both in the natural world and in animal models, where it is much easier to create a goat model than a mouse model of AF, for example. This also extends to humans—athletes, taller people, and obese individuals all have large atria and are more likely to exhibit AF. Within an individual, risk factors such as hypertension, valvular disease and ischaemia can enlarge the atrium and increase the risk of AF. With respect to AF ablation, we explore how variations in ablation strategy and the relative effectiveness of these strategies may suggest that a reduction in electrical atrial mass is an important mechanism of action. We counter this with examples in which there is no doubt that mass reduction is less important than competing theories such as ganglionated plexus ablation. We conclude that, when considering future strategies for the ablative therapy of AF, it is important not to discount the possibility that contiguous electrical mass reduction is the most important mechanism despite the disappointing consequence being that enhancing success rates in AF ablation may involve greater tissue destruction.

## Introduction

Since the work of Garrey^1^ in 1914 it has been accepted in the field of cardiac electrophysiology that size matters in the aetiology of fibrillatory arrhythmias. Atrial fibrillation (AF) ablation clearly reduces the electrically contiguous mass of the atrium. Despite this, however, electrical mass reduction is not generally accepted to be an important part of the mechanism by which ablation reduces arrhythmia frequency.

Whether in the basic science lab or in a clinical case of a patient with mitral valve disease, we all intuitively understand the importance of atrial size in the aetiology of AF. We even accept that a larger atrium is likely to make it more difficult to treat AF, whether by ablative or other means. When it comes to theories regarding the mechanism of action of AF ablation, however, atrial mass reduction is usually considered subsidiary to other mechanisms such as targeting of ectopy triggers, fractionated signals, rotational activity and the cardiac autonomic nerve network.

The goal here is to ensure that investigators might consider that size matters in AF ablation, just as we all know it does in AF pathogenesis. Of course, accepting this proposition has the somewhat disappointing consequence that enhancing success rates in AF ablation may necessarily involve greater tissue destruction. It is also the case that we cannot draw firm conclusions regarding the importance of this mechanism as compared to the others mentioned and we counter this theory with examples of patients in whom other mechanisms are undoubtedly of greater importance. However, we hope this review accompanied by our perspectives provides readers an expanded understanding of this concept, which can then be incorporated into future investigative strategies in the field of AF ablation.

## Animal and tissue models show that size matters in AF

### Early experimental work

Garrey^1^ first outlined this in a series of experiments published in 1914. It is valuable to quote the original text here: ‘When the auricles of cats, rabbits, or dogs are stimulated with strong faradic shocks…auricles enters into violent fibrillary contractions…it was found that when a portion of the wall of fibrillating auricles was picked up by forceps and functionally separated from the heart by ligating, or by clamping with haemostatic forceps, the portion so separated ceased fibrillating…although the organ from which it was removed continued its incoordinated contractions unaltered’.^1^

Further work by Garrey exploring AF mechanisms in experimental models showed that AF was also more efficiently induced in larger tissue masses.^2^ He also describes dividing the atrium into two larger segments in which case fibrillation sometimes continues in both large segments concluding that this is evidence against a ‘focal tachysystolic pacemaker’. Below a certain size, however, he finds that atrial tissue simply cannot sustain fibrillatory activity. As such there was clearly a well-formulated critical mass hypothesis over a century ago.^1^^,^^2^

### Evidence of variation in ability to induce AF by animal size and tissue dimensions

Atrial size corresponds to body surface area,^3^ and it had been thought that small rodents were incapable of sustaining AF.^4^ The ‘critical mass hypothesis’ was founded and widely supported, with estimated minimum chamber sizes required of approximately 100–200 mm^25^ and 400 mm^21^ for AF and ventricular fibrillation (VF), respectively. In 1994, Winfree^5^ discussed the computational and experimental evidence that showed while rotational activity can occur in very thin cardiac slices, for tissue to fibrillate, the third dimension is a pre-requisite. The thickness threshold was defined as 1/π times the distance a spiral wave propagates during one rotation period.

Along similar lines, AF is easier to induce in larger animals than small animals. Atrial surface area, a correlate of atrial enlargement, has been found to be independently associated with risk of AF in preclinical models. In domestic pigs, increasing atrial surface area coincided with a reduction in the atrial effective refractory period (ERP), and both variables were independently associated with probability of sustained AF on logistic regression analysis.^3^ Similar findings have also been reported in canine models.^6^ Moreover, in veterinary medicine, AF is prevalent in large animals, such as horses^7^ and whales,^8^ and is more common in large-breed than small-breed dogs.^9^

**Figure 1:**
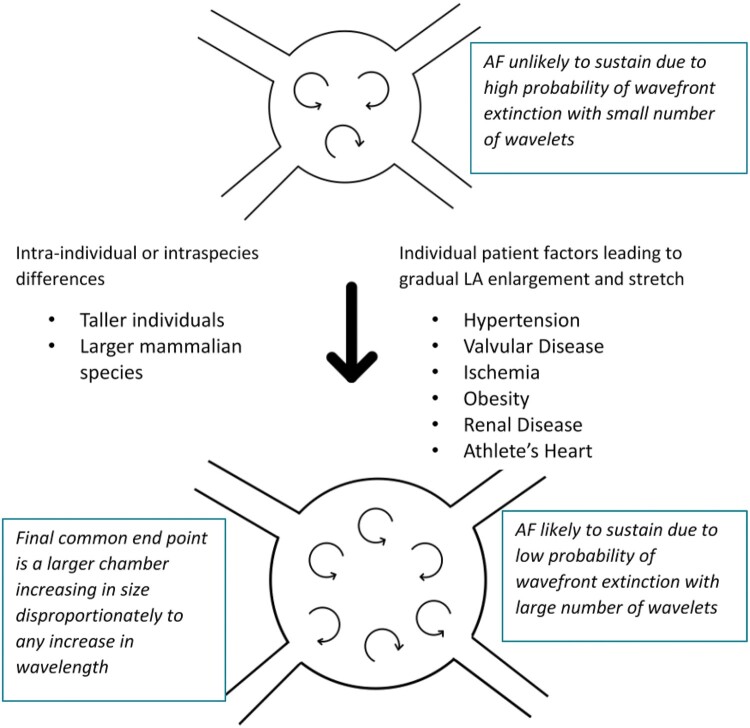
Larger atria are more likely to exhibit atrial fibrillation. If wavelength of atrial tissue is represented by the arrow length, it is logical that if atrial size increases out of proportion to wavelength, more simultaneous wavelets can be simultaneously present in the atrium, vastly reducing the chance of spontaneous termination. AF, atrial fibrillation; LA, left atrium; LV, left ventricle.

There is significant evidence therefore in preclinical models, as well as the animal kingdom, that the ability to sustain AF is heavily dependent on atrial size. One simple explanation, but one which has not been superseded, is the combination of two important physiological details.^10^ Firstly, even when discounting increasing atrial wall thickness, the maximal number of activation wavefronts in the atrium increases with the square of the atrial diameter, and secondly, in larger mammals the wavelength of the atrial impulse does not increase proportionally to the size of the atria. This means that in larger mammals more simultaneous fronts of depolarization can propagate simultaneously, reducing the likelihood of arrhythmia termination. This was recognized by Moe in 1959 who stated: ‘If the number [of wavelets] is large, there is little chance that all elements will fall into phase but if the number is small there is a considerable probability that they may fuse and permit resumption of sinus rhythm…obviously a large mass of tissue can support a larger total number of independent wavelets’.^11^ This is illustrated in *Figure 1*.

This is an extreme simplification of a much more complex reality of course. Weiss *et al*.^12^ have expanded on these concepts looking at the important role of wavebreak in fibrillation. This describes a situation where, instead of orderly single waves of depolarization, the leading edge of one wavefront interacts with the trailing edge (waveback) of another. Re-entry can begin at a localized wavebreak since the wavefront is highly curved at this point, sometimes resulting in a rotor.^13^ This is made more likely by heterogeneous tissue, for example in the situation where a wavefront encounters tissue with a longer refractory period than surrounding tissue. This does not change the basic premise, however, that there must be sufficient atrial tissue for these mechanisms to result in sustained, simultaneous activity of multiple wavefronts.

As such, it was widely thought that murine models would not be feasible experimental models of fibrillation. However, Vaidya *et al*.^14^ demonstrated VF in Langendorff-perfused normal adult mouse hearts for the first time in 1999, with chamber areas of >100 mm^2^, challenging the thoughts on minimum chamber size required for fibrillatory arrhythmias. Since then, multiple rodent experimental models of AF have been developed, but often these require significant genetic modification.^15^ In larger animals such as sheep, dogs and goats on the other hand, AF can be induced in models that replicate human causes of AF such as rate-related remodelling (rapid atrial pacing), volume overload (mitral valve rupture), hypertension, myocardial infarction and pericarditis. In the mouse, by contrast, *in vivo* models revolve around gene knockouts or overexpression, often of ion channel, sarcomeric proteins, or of pathways that up-regulate fibrosis. With such major genetic changes, mice can be induced to have AF. Rats are less amenable to genetic modification, and attempts to model AF through acquired disease such as myocardial infarction have not resulted in electrocardiographic evidence of AF.^15^ The spontaneously hypertensive rat has been used as a model of AF. Here though, AF is not spontaneous, and aggressive burst pacing is required to induce AF.^16^

### How does AF beget AF in animal models—electrical vs. structural remodelling

In 1995, Wijffels *et al*.^17^ investigated the specifics of atrial remodelling in a goat model in which AF was induced by 50 Hz burst pacing with atrial electrodes. At baseline, AF stimulated in this way in goats only lasted a few seconds and terminated spontaneously, but with repeated stimulation of AF over several days or weeks, persistent AF could be maintained with no further atrial pacing bursts. Thus, for the first time it was demonstrated that burst-pacing inducing AF leads to atrial remodelling that is pro-fibrillatory: AF begets AF.

What does this type of model tell us about the importance of chamber size in AF begetting AF? Firstly, it is very clear that this adverse remodelling is not all size dependent. In the first few hours there is significant electrical remodelling with the AF cycle length shortening at a rate of 1-2 ms/h.^18^ By 4–6 days, a steady state is reached with no further shortening of AF cycle length. In a follow-up study, Wijffels *et al.* showed that the reduction in refractory period in this model in the acute phase was probably not due to acute atrial stretch—they did so by showing no change in atrial ERP with acute IV volume infusion in goats.^19^

The time course of reverse electrical remodelling after restoration of sinus rhythm should also be considered.^17^^,^^20^^,^^21^ It is informative to note that even after prolonged periods of AF (months to years) in both humans and goats, within days the changes in atrial ERP are completely reversed. Therefore, late recurrences in AF (e.g. more than a week after cardioversion) cannot be explained by adverse electrical remodelling.

Later experiments look more closely at the electrical and structural changes important in these models. There appear to be different phases in the remodelling. In mongrel dogs it was shown that in the first few hours of rapid atrial pacing, there is a significant autonomic-dependent component to the electrical remodelling, with the reduction in ERP and vulnerability to AF with pacing protocols being abrogated by ablation of cardiac autonomic nerves.^22^ Whilst electrical remodelling reaches steady state within 3–4 days, it often takes weeks for AF to become persistent such that spontaneous termination of AF ceases to occur in such models.^17^ Over time there are significant structural changes, particularly in terms of an increase in atrial cell size, perinuclear accumulation of glycogen and extracellular matrix protein accumulation.^20^^,^^23–29^ These changes are less easily reversible and are associated with an overall enlargement of the atrium by echocardiographic and gross histological measures.^28^^,^^29^ These less reversible changes also correspond with the time period in which AF becomes persistent, underlining the importance of these structural changes.

Atrial fibrosis may be a particularly important aspect of both atrial structural and electrical remodelling in AF.^30^ Fibroblasts make up 75% of cardiac cells by number and can be electrically coupled to cardiac myocytes.^31^ Proliferation of fibroblasts would certainly increase atrial size but can also alter conduction properties and heterogeneity of tissue. Fibroblasts can produce large quantities of matrix proteins which block conduction. By way of their coupling to myocytes, they can also enhance heterogeneity of repolarization, resulting in an increased probability of re-entry.

It is also clear from experiments involving techniques which cause atrial enlargement, such as atrioventricular block and cardiac failure models, that there is an interplay between such structural remodelling and the ability of a final trigger to induce AF.^32^^,^^33^ AF episodes are more prolonged at an earlier stage of the AF induction models after a prior phase of adverse structural remodelling, even when that structural remodelling was not caused by AF itself.

## Computational modelling—size matters in AF aetiology and ablation strategy

The critical mass hypothesis, as discussed earlier, states that there are minimum chamber sizes required to support fibrillatory arrhythmias, with greater chance of wavefront extinction in smaller tissues. This hypothesis has been supported by a number of computational simulations of fibrillation, predominantly in simulations of multiwavelet re-entry. In these studies, it has been demonstrated that larger tissue masses promote wavebreaks, multiwavelet re-entry and therefore fibrillation.^34^ The fibrillogenicity index, predicting the duration of multiwavelet re-entry episodes, was established in another *in silico* study, with tissue area being a significant component of the formula.^35^

One of the aims of therapeutic ablation for AF is to increase the likelihood of fibrillatory wavelets encountering non-conductive tissues/barriers and thus terminating. Computational modelling studies have explored various ablative strategies, and identified that the chance of multiwavelet re-entry termination is directly related to the ratio of boundary length to tissue area.^36^ Therefore, greater compartmentalization of a tissue mass provides an increased prospect of wavefront annihilation in AF sustained by multiwavelet re-entry. The same group have also ascertained that ablation involving areas of high circuit-density (that is regions with multiple coexisting dynamic circuits) most efficiently reduces re-entrant wave duration,^37^ and they have also refined an individualized lesion set that is necessary to effectively minimize multiwavelet re-entry.^38^ This concept has been taken forwards in a randomized study of patients with persistent AF, reporting that *in silico* modelling guided ablation was non-inferior to an empirical lesion set, in terms of procedure time, ablation time, major complication rate as well as AF recurrence rate at 12 months.^39^

## Human data regarding chamber size in AF causation

### Human height and weight are correlated with AF propensity

Just as in other mammals, larger humans are predisposed to AF development. Obesity has been identified as an AF risk factor in clinical prospective cohorts, which in part may be attributable to differences in left atrial (LA) dilatation between obese and lean individuals. Increased body mass index (BMI) is an important indicator of LA size, which may be driven by various mechanisms—activation of the renin–angiotensin–aldosterone system, ventricular stiffening and diastolic dysfunction, as well as increased plasma volume and myocardial injury by oxidative stress.^40^ The association between BMI and AF was found to be independent of blood pressure and hyperlipidaemia, and only partially dependent on the presence of diabetes, in multivariate analysis in a population-based case-control study.^41^ This risk factor for AF, however, is modifiable with >10% weight loss in overweight individuals resulting in a six-fold greater probability of arrhythmia-free survival, compared to those patients who do not lose weight.^42^ Larger body surface area in youth is also independently associated with the propensity for future AF development,^43^ whilst taller individuals are likely to have larger atria, and also are at increased risk of AF.^44^ A low incidence of AF in individuals of South Asian ethnicity has been reported, despite a high prevalence of conventional cardiovascular and AF risk factors. Plausibly, this may be due to smaller atria in South Asian individuals, related to smaller body surface area.^45^

### Aerobic fitness, atrial size, and AF

There is a U-shaped association between exercise and AF with levels of physical exercise. Both being very sedentary and participating in high-level competitive endurance sport are risk factors for AF.^46^ In long-distance cross-country skiers, those with faster finishing times and more completed races were found to be at increased risk of arrhythmias, mainly driven by AF.^47^ A meta-analysis of case–control studies has shown that athletes are at modestly increased AF risk with a point estimate for hazard ratio of 2.34,^48^ whilst a retrospective cohort study demonstrated that LA diameter and volume were strong AF predictors in men undertaking long-term endurance sport.^49^ There does also appear to be a dose-response relationship for endurance training and AF risk, with the highest risk coming after many years of endurance athletic training and extending into retirement of these athletes.^50^^,^^51^

Where athletes are taller, they are also at higher risk for AF than their shorter athlete counterparts. A retrospective analysis of Swedish military conscripts showed that the combined risk of height and aerobic fitness was greater than the sum or product of the individual risks, suggesting an important compounding interaction between these risk factors.^52^ Much has been made of the heritability of AF, which was 22% in a recent large genome-wide association study,^53^ but height and weight are highly heritable also—it is unclear how much of the heritability of AF might be attributed to these much more phenotypically obvious attributes.

### Comorbidity, atrial size, and AF propensity

Other aspects of human health and disease have the potential to affect LA size. Systemic hypertension, in part via diastolic dysfunction, leads to LA enlargement. Valvular heart disease, especially mitral valve disease, often leads to significant LA enlargement. In mitral stenosis, LA enlargement and AF development is strongly associated with reduced survival.^54^ Other structural heart conditions, such as congenital heart defects, cardiac masses and systemic arteriovenous fistulae can also modulate LA size.^55^ Coronary ischaemia, with and without left ventricular systolic impairment, can similarly result in LA enlargement.^56^ Non-cardiovascular diseases associated with AF, including renal disease and diabetes, are also known to increase LA size.^57^

A useful integrative measure of the amount of adverse atrial remodelling that has occurred in an individual is the LA volume as measured by echocardiography or other imaging techniques. This is both a useful predictor for future AF development^58^^,^^59^ and for some of the adverse consequences of AF, including stroke and death.^60^^,^^61^

In patients with co-morbid conditions leading to AF, it is important to state that atrial enlargement is not the only important factor. For example, in a case series of patients with rheumatic valvular disease, mitral stenosis was associated with a higher prevalence of AF (29%) than mitral regurgitation (16%) despite a similar increase in LA dimension.^62^ Continuous pressure overload on the left atrium in mitral stenosis was thought to explain this difference.

### Importance of atrial size in the transition from paroxysmal to persistent AF

Above, we outlined the importance of adverse electrical remodelling in animal models of AF. In patients with no prior AF, brief 15-minute induced episodes are sufficient to result in similar electrical remodelling as well as lowering the threshold for inducibility of AF.^63^

**Figure 2:**
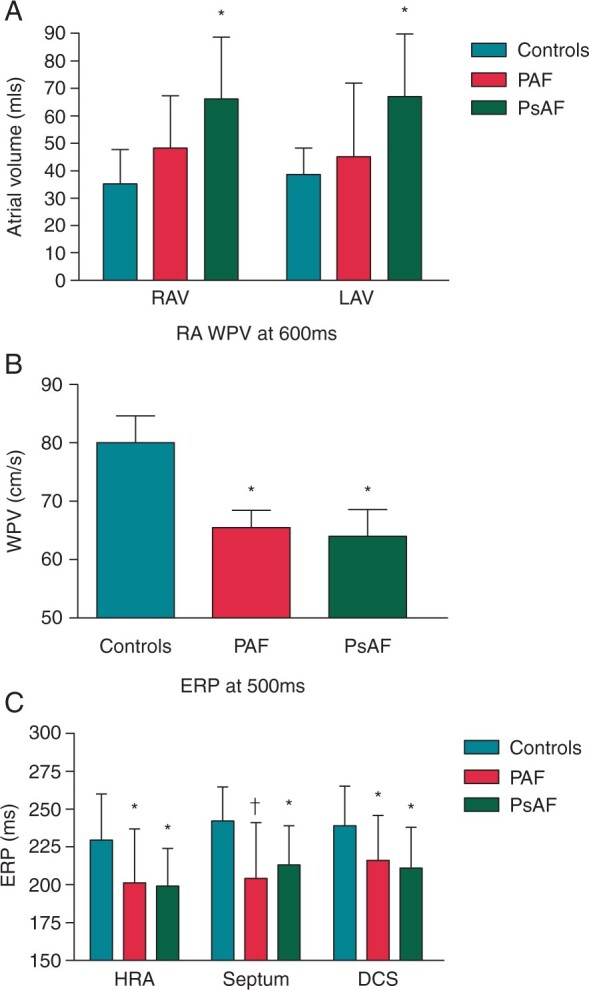
Increase in atrial size is more important than electrophysiologic remodelling in progression from paroxysmal to persistent AF. Reproduced with permission from Kojodjojo *et al.*^67^ (*A*) Right atrial volume (RAV) and left atrial volume (LAV) progressively increase from control patients to those with paroxysmal AF (PAF) with a further increase in those with persistent AF (PsAF). (*B*) Wave propagation velocity is reduced in both PAF and PsAF to a similar extent. (*C*) Effective refractory period (ERP) is reduced in both PAF and PsAF to a similar extent. Various locations showed similar findings including high right atrium (HRA), septum and distal coronary sinus (DCS).

Similar to animal models, in patients with AF, there is a shorter ERP^64^ and absence of its rate-dependent adaptation.^65^ Moreover, spatial variability of refractoriness is enhanced in AF patients,^66^ whilst slower conduction has also been associated with propensity to AF.^64^ Kojodjojo *et al.*^67^ reproduced these findings in a study assessing conduction velocity and refractory periods in patients undergoing LA ablation for left-sided accessory pathways vs. those having AF ablation for paroxysmal AF and those with persistent AF. A key finding was that, although there was global shortening of refractoriness and conduction slowing in patients with AF, these properties did not differ between patients with paroxysmal and persistent AF, and the only difference between these two groups was chamber size, which was more dilated in the persistent group. This makes the important point that further remodelling in persistent AF is more related to chamber size than electrophysiological properties (*Figure 2*).

These findings are supported by the observation that LA enlargement and longer duration of AF make sinus rhythm maintenance less likely almost regardless of which rhythm control strategy is employed, be it cardioversion, medication or ablation.^68–71^ Longer episodes of AF also result in slower return of full atrial contractile function post-cardioversion. This can take up to one month in AF episodes of greater than 6 weeks duration.^72^

## How does this relate to AF ablation strategy?

### Competing theories regarding mechanism of action of AF ablation

The cornerstone of AF ablation is pulmonary vein isolation (PVI). The success rate for PVI is highly variable, from around 40–100% for paroxysmal AF at 1 year.^73^^,^^74^ We have shown that this variation depends to a large extent on the methodology of detecting recurrence and the patient mix (rather than the specific methodology of PVI).^75^ Certainly PVI is the most effective element of the various ablation strategies used thus far, reducing AF recurrence in comparison to strategies where PVI was not performed by 54% in a recent meta-analysis.^76^

Presence or absence of a recurrence, however, is a poor measure of the overall success of this procedure. A recent study incorporating implanted loop recorders in all patients pre- and post-ablation showed a ‘success rate’ of 53% in terms of absence of recurrence of AF post-PVI within 1 year. When analysed in terms of the arguably more clinically relevant total burden of AF, there was a reduction of >98% post-PVI compared to pre-PVI.^77^

While the effectiveness of PVI is not in doubt, the mechanism of its effect certainly is debated. The original theory of ectopy from the pulmonary vein (PV) sleeves being prevented from exiting into the atrium^78^ (a pure trigger-prevention theory), whilst an initially important paradigm, has since been shown to be an oversimplification. Other theories relate to modification of ganglionated plexi (GP)—in a probability mapping study, we have shown that these are often peri-venous in the left atrium and likely to be modified by collateral damage during ablation procedures, particularly adjacent to the right upper PV.^79^ Basket electrogram mapping studies suggest that the PV antral regions have a particular propensity for rotational and focal activity, and so ablation of these regions may limit such activity, thus reducing the likelihood of AF initiation and maintenance.^80^

There is no doubt that the PVs differ from the remainder of the atrium electrically. A seasoned electrophysiologist can identify PV signals very rapidly from their sharpness and amplitude relative to other locations in the atria. This is rooted in differences in electrophysiology. Embryologically the PVs and posterior wall have a different origin than the primary heart tube and systemic veins, coming from a midpharyngeal strand which develops into mediastinal myocardium at about 6 weeks gestation.^81^ On the cellular level, PV myocytes are more likely to have spontaneous phase 4 depolarizations and have reduced density of I_k1_ channels leading to reduced stability of the resting membrane potential.^82^ On the tissue level, PV myocardium has a shorter ERP and anisotropic conduction (which is unidirectionally slowed in the LA to PV direction).^83^^,^^84^ These changes increase the chance of re-entry in the vicinity of the PVs. As such, it would be simplistic to propose that PV isolation is simply a debulking strategy.

### Could reduction in electrically active atrial mass be relevant?

As well as the other proposed mechanisms of action, though, PVI can substantially reduce the electrical mass of the left atrium. The myocardial sleeves in the PVs can extend 2.5 cm out of the atrium, and in veins with a diameter of approximately 2 cm and with the antrum isolated as well, with four veins isolated during each ablation, this is a substantial proportion of the atrial mass that is rendered electrically inactive.^85^ Going back to Winfree’s postulation of the essential nature of the third dimension for fibrillatory wavefronts,^5^ some of the regions ablated during PVI have substantial thickness—averaging 4.4 mm at the left lateral ridge^81^ compared with closer to 2 mm in terms of average atrial wall thickness in many studies.^86^

There is also a greater PV mass in AF patients. Hassink *et al.*^87^ found it more likely that PV sleeves were present in post-mortem specimens of patients with AF than those without. PV ostial dilatation is also more often found in imaging studies of patients with AF than patients who have not had AF.^88^ Moreover, PV myocardial tissue has been found to be thicker in patients with AF via intravascular ultrasound.^89^ Whether or not these changes were present before patients went into AF or are simply related to adverse remodelling is unknown. Regardless of the time sequence however, these anatomical changes could be an important part of the pathophysiology of AF and the increasing propensity to persistent AF over time with adverse atrial remodelling.

There is some evidence that this mass-reducing hypothesis is an important component of AF ablation success. Early work by Pappone *et al.*^90^ showed that the encircled area of the atrium incorporated in a PVI was the only element significantly associated with procedural success, although perhaps the denominator (bigger atrial area associated with lower success) may have been a more important factor than how wide the ablation was. Oral *et al*.^91^ showed that wider encirclement of the veins was more effective than ostial isolation, which has now become standard practice. In addition, leaving a small gap in the ablation line results in a lower procedural success, suggesting that reduction in contiguous atrial mass is an important part of successful ablation,^92^ although this is also consistent with the regional driver hypothesis.

The evidence that atrial electrical debulking is important in the mechanism of action of AF ablation is equally compelling for the cryoballoon technologies. The second generation cryoballoon is an effective ablation tool and mapping studies have shown that this technique successfully ablates not only the PV sleeves but also on average 73% of the surface of the posterior LA wall.^93^ The fact the 28 mm (rather than 23 mm) cryoballoon has been widely adopted may in part relate to the enhanced debulking this technology provides.^94^

### What can ablation strategies other than PVI teach us about the importance of the mass reduction hypothesis

In terms of alternative ablation strategies, those that reduce contiguous atrial mass less than isolating a complete anatomical structure, seem to be less effective than those that do. Complex fractionated atrial electrogram (CFAE) ablation and linear ablation lesions exemplify this. Both these strategies can set-up an increased risk of atrial tachycardia (AT).^95^^,^^96^ Wong *et al.*^95^ showed a net neutral effect on overall AT/AF recurrence by adding CFAE to PVI and linear ablation. A closer look as to the mode of recurrence showed a higher recurrence rate of AT and lower recurrence of AF in the CFAE arm. Hence the atrial debulking related to CFAE can be useful in reducing fibrillation as would be expected within the framework of a mass reduction hypothesis, but the strategy will often result in an atrial substrate conducive to ATs in a way that more contiguous debulking strategies (e.g. posterior wall and LA appendage isolation—see below) do not. By forming islands of non-contiguous lesions, CFAE can lead to areas of slow conduction, reducing wavelength such that a wider excitable gap exists, pre-disposing to macro re-entrant flutters. This is particularly the case in the context of gaps in linear lesions.

In a high-quality randomized study, CFAE and linear ablation were shown not to improve success in the ablation of persistent AF compared to PVI alone,^97^ and there was a trend towards reduced success with these additional lesion sets. Other strategies aiming to target areas of focal and rotational activity without necessarily performing PVI, have had variable success rates when studied by different groups.^98–100^ Thus, there seems a general trend that specifically targeting regions of the atrial myocardium with special electrophysiologic properties during ablation, is less consistently successful than strategies that are not as targeted and specific, and more related to regional isolation and functional atrial debulking—PVI, posterior wall isolation, and appendage isolation.

**Figure 3:**
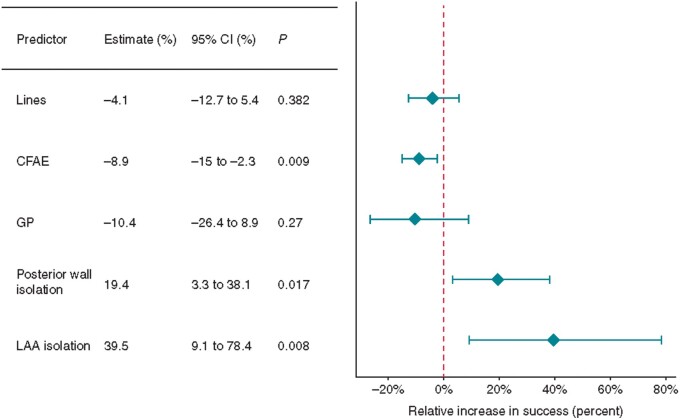
The effect of procedural predictors on freedom from atrial fibrillation. Reproduced with permission from Sau *et al*.^101^ AF, atrial fibrillation; CFAE, complex fractionated atrial electrogram; CI, confidence interval; GP, ganglionated plexi; LAA, left atrial appendage.

The authors’ contribution to thinking about an optimal lesion set in persistent AF was a recent meta-regression analysis looking at the procedural variables predicting success in a range of ablation studies incorporating 6767 patients with persistent AF. In terms of lesion sets that would predict success, we showed that lines, CFAE and GP ablation were not associated with improved outcomes, however isolation of additional anatomical structures such as the posterior wall and LA appendage seemed to predict higher procedural success (*Figure 3*).^101^ This is consistent with reduction of atrial mass as an important mechanism in AF ablation. In the same study, we also found that larger LA size was associated with lower success rates, similar to a previous multivariate analysis of predictors of persistent AF ablation success.^102^

Surgical data appears to mirror that of percutaneous ablation. Each iteration of the Cox-Maze procedure has incorporated PVI as well as posterior wall isolation.^103^ When the box lesion was left out, for example by not performing a posterior line, outcomes have been significantly worse.^104^ Similar to catheter ablation, large LA size is associated with greater AF recurrence and can predict treatment failure.^105–107^

### Arguments against the importance of critical mass reduction in mechanism of action of PVI

We would not wish to leave readers with the impression that critical mass reduction is the final word in the mechanism of action of AF ablation. Arguments against this theory are valid and deserve further discussion.

As noted above, the remodelling that happens in the acute phase in pacing induced models and already starts to enhance the duration of paroxysmal AF, has been shown to be overwhelmingly electrical rather than structural, such that size alone cannot be the explanation for all forms of AF.^19^ Similar arguments can be made for other forms of acute metabolic or hormonal perturbations (e.g. electrolyte abnormalities, thyrotoxicosis) in otherwise healthy atria. An enlarged, adversely structurally remodelled LA is not the *sine qua non* of AF.

It is also true to say that the other mechanisms above are important, the fact that GP ablation, for example, can abrogate the electrical changes in pacing induced models, tells us that in a sub-set of AF, GP ablation is likely the only mechanism required to treat AF without having to invoke a mass reduction hypothesis.^22^ We have certainly observed AF ablation cases in patients where GP stimulation results in incessant AF resistant to cardioversion,^108^ and have exemplar patients where ablation of very limited regions of ectopy triggering GPs is sufficient to render patients AF-free for over a year without needing to perform PV isolation.^109^ Targeting of specific focal and rotational activation patterns using basket catheters has also been shown in limited numbers of cases to be able to acutely terminate AF, usually by targeting just one to three sites of such activity.^110^ In one case report, simple pressure on a site of highly fractionated signals remote from the PVs reproducibly and recurrently resulted in termination of persistent AF.^111^ Isolation of parts of the atrium which persist in AF while the remainder of the atrium reverts to sinus rhythm, e.g. the LA appendage as described by Wu *et al.*,^112^ also add to the arguments against electrical mass reduction explaining the totality of effectiveness of PVI. When such limited ablation results in long-term sinus rhythm, this is an ideal result. The overarching aim of individualised catheter ablation is to permit sinus rhythm maintenance with the least amount of myocardial scarring and compromise of atrial function.

Hopefully the above evidence makes it clear that this is not an argument that PVI is functioning via an electrical mass reducing effect in all cases. Instead, our argument is that it is an underrecognized contributor to the mechanism of action of PVI in a large proportion of AF ablation cases.

## Conclusion

The importance of atrial size in AF was proven experimentally over 100 years ago. Despite this, the reduction of electrically active atrial mass that occurs during AF ablation is underrecognized as an important part of its mechanism of action.

We have shown the importance of atrial size in the aetiology of the condition. Animal models of AF are more easily induced and are more clinically relevant in larger mammals, and an important element of these models is the cellular and tissue remodelling which leads to LA enlargement. Naturally occurring AF in the animal kingdom is also more prevalent in larger mammals. In humans, a wide range of predisposing factors and illnesses with a common endpoint of an enlarged LA are associated with an increased risk of AF. These include being tall, obese, or athletically very fit as well as the common associations with hypertension, valvulopathies and coronary disease.

We have shown that AF ablation techniques substantially reduce contiguous electrical mass and that when looking at the totality of available data (in a large meta-regression analysis), the most effective ablative strategies in persistent AF are those that reduce contiguous electrical mass most effectively. On the other hand, we have also shown that competing theories of how AF ablation produces its clinical effect have merit and are significant in some patients. Moreover, LA size impacts therapeutic decision making, with a lower likelihood of successful cardioversion and reduced suitability for catheter ablation with larger masses.

In conclusion, when considering future strategies for the ablative therapy of AF it is important not to discount the possibility that contiguous electrical mass reduction is the most important mechanism of action, despite the somewhat disappointing consequence of this being that enhancing success rates in AF ablation may necessarily involve greater tissue destruction.

## Funding

A.H. is funded by a Wellcome Trust Clinical Research Fellowship (Grant number 220572/Z/20/Z).

**Conflict of interest:** none declared.
